# A Quadrilateral Geometry Classification Method and Device for Femtocell Positioning Networks

**DOI:** 10.3390/s17040817

**Published:** 2017-04-10

**Authors:** Jeich Mar, Tsung Yu Chang, Yu Jie Wang

**Affiliations:** 1 Department of Communications Engineering, Yuan-Ze University, Taoyuan 320, Taiwan; k3299789@gmail.com (T.Y.C.); a3020894@gmail.com (Y.J.W.); 2 Communication Research Center, Yuan-Ze University, Taoyuan 320, Taiwan

**Keywords:** femtocell positioning network, optimum geometry disposition decision criteria, normalization multi layer perception, iterative geometry training algorithm, time difference of arrival, cloud computing platform

## Abstract

This article proposes a normalization multi-layer perception (NMLP) geometry classifier to autonomously determine the optimal four femtocell evolved Node Bs (FeNBs), which can use time difference of arrival (TDOA) to measure the location of the macrocell user equipment (MUE) with the lowest GDOP value. The iterative geometry training (IGT) algorithm is designed to obtain the training data for the NMLP geometry classifier. The architecture of the proposed NMLP geometry classifier is realized in the server of the cloud computing platform, to identify the optimal geometry disposition of four FeNBs for positioning the MUE located between two buildings. Six by six neurons are chosen for two hidden layers, in order to shorten the convergent time. The feasibility of the proposed method is demonstrated by means of numerical simulations. In addition, the simulation results also show that the proposed method is particularly suitable for the application of the MUE positioning with a huge number of FeNBs. Finally, three quadrilateral optimum geometry disposition decision criteria are analyzed for the validation of the simulation results.

## 1. Introduction

The latest Long Term Evolution-Advanced (LTE-A) standard anticipates the increasing use of small cells (known as femtocells) to provide the geo-location information required to meet the emerging communication and networking needs of future smart city applications [1,2]. An overview of the LTE positioning methods is provided in [3], which includes the analysis of previous indoor localization methods. The aim of the paper [3] is focused on the investigation of the floor detection techniques in indoor environments. Based on the massive multiple-input multiple-output (MIMO) antennas and the millimeter wave communication technologies, the small cell concept will appear on the fifth generation (5G) cellular networks [4]. To realize the seamless coverage, a larger number of small cells have to be densely deployed in Heterogeneous network (HetNet) scenarios. This trend results in an attractive technological study of LTE outdoor localization. In providing location-specific services, the location of the moving macrocell user equipment (MUE) must be known with a high degree of accuracy. Then, the FeNBs can steer the digital beam-forming (DBF) to suppress the interfering signal generated from the uplink transmission of the outdoor MUE. Additionally, it could allow the call of the MUE to be handed to the indoor FeNBs, thereby improving the system efficiency.

MUE localization is conventionally performed using a standalone GNSS/GPS system [5]. However, such systems not only incur a high battery consumption, but may also lack the precision required to provide true location-specific services. In some cases (e.g., indoor environments or built-up urban areas), the GPS signal may be intermittent or too weak to perform localization. Notably, the GPS measurements obtained using the MUE cannot be directly used for localization purposes in femtocell networks, since they cannot be transmitted in real time to the femtocell evolved Node Bs (FeNBs) [6]. Various methods have been suggested for improving the MUE localization performance in wireless networks [7,8]. Thus, in a more recent 3GPP specification (Release 11) [9], it is proposed that MUE positioning be performed by the radio access net-work (RAN) itself, rather than the MUE.

Existing MUE positioning methods, including those based on time difference of arrival (TDOA), direction of arrival (DOA), or hybrid TDOA / DOA measurements, generally utilize a Gauss-Newton Interpolation (GNI) algorithm to estimate the location of the MUE in two-dimensional (2D) space [8,10]. Multi-station TDOA positioning systems use optical communications to send the received signal of each FeNB to a reference FeNB, which then uses this information to calculate the TDOA between itself and each transmitting FeNB. Such systems have the advantages of a high accuracy and low system complexity. Furthermore, the common channel error among the different FeNBs is easily eliminated, and thus, the synchronization problem becomes trivial.

TDOA positioning systems require at least four FeNBs to locate the moving MUE in three-dimensional (3D) space. However, the accuracy of the localization results depends on the geometry disposition of the selected FeNBs. The geometric dilution of precision (GDOP) contour map of four stations is simulated in [11]. It shows that the square distribution shape provides the best coverage area, the lozenge shape is the second best, the other irregular quadrilateral (IQ) shapes are the third best, and the straight line has the worst coverage area. Still, to the best of the author’s knowledge, there is no other optimization approach provided to autonomously determine the optimum geometry disposition of four FeNBs. Accordingly, the present study proposes a normalization multi layer perception (NMLP) geometry classifier to autonomously determine the optimal four FeNBs with a lower GDOP value and an iterative geometry training (IGT) algorithm to obtain the training data for the NMLP geometry classifier. The most well-known neural network is the MLP architecture [12], which is widely used for solving problems related to the classification of the different patterns. Normalizing the inputs can make training faster and reduce the chances of getting stuck in local optima with the exact same outputs that the MLP had before [13]. Having obtained the optimal set of FeNBs, the position of the MUE is estimated using the TDOA method and updated using a tracking filter (such as an extended Kalman filter or an adaptive α-β-γ filter [14], which will not be included in this article), as soon as the MUE transmission cannot be continuously received.

In addition, three optimum geometry disposition decision criteria are analyzed for the square, lozenge, and other quadrilateral shape of the four FeNBs for positioning the moving MUE when two or more of the same quadrilateral shapes are determined. The derived analytical expressions are generally applicable to geometries where the MUE is surrounded by the selected FeNBs. A comparison of the analytical results with simulations using the typical geometries of outdoor positioning systems shows good agreement.

The structure of the paper is described as follows. Section 2 presents the femtocell positioning network model. The architecture of the NMLP geometry classifier and the IGT procedure for generating the training data are described in Section 3. In Section 4, three optimum geometry disposition decision criteria are also considered, to determine the optimum shape of four FeNBs in the square, lozenge, and irregular quadrilateral, respectively. In Section 5, the performance of the proposed NMLP geometry classifier is simulated in the outdoor geo-location scenario. It consists of 16 FeNBs located in two adjacent multi-floor buildings. Finally, Section 6 concludes the paper.

## 2. Femtocell Positioning Network Model

**Figure 1:**
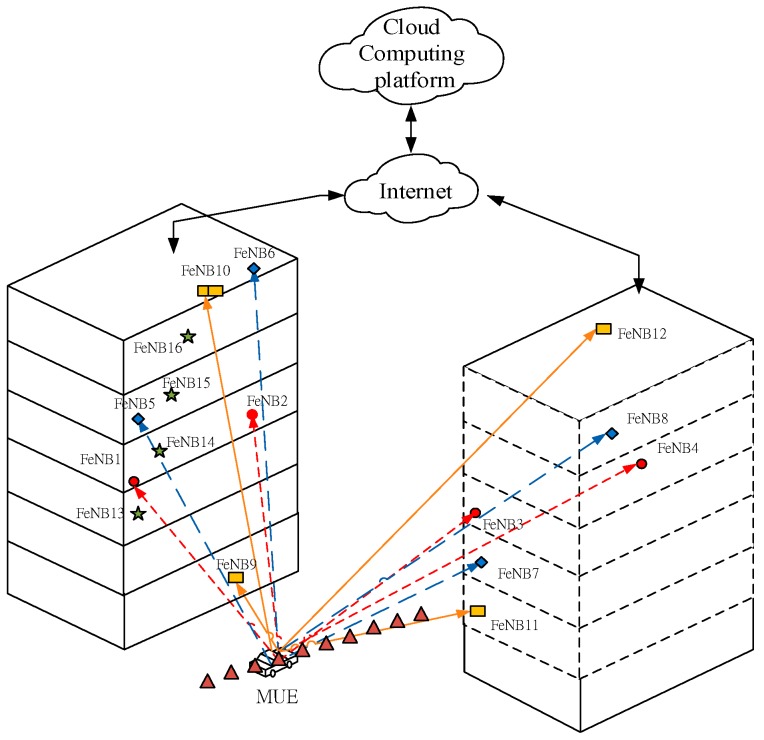
Outdoor geo-location scenario between two multi-floor buildings.

The scenarios under study include the combination of a macro cell with femtocells in outdoor environments, which are operated at the same frequency band. It is assumed that the interference coordination and smart digital beam forming techniques may be needed to solve the problems of co-channel interference and signal attenuations. Figure 1 shows an illustrative femtocell network consisting of multiple FeNBs deployed throughout two adjacent multi-floor buildings. An assumption is made that the FeNBs are connected to a cloud-computing platform through the Internet. Moreover, the coordinates of each FeNB and the training data are pre-stored in the database of the cloud-computing platform. The training data of the NMLP geometry classifier are determined using an IGT algorithm based on the TDOA measurements obtained by each FeNB and the GDOP metric. The architecture of the NMLP geometry classifier is realized in the server of the cloud-computing platform to perform the searching of the optimal set of FeNBs for positioning purposes.

The uplink transmissions from the MUE are conventionally handled using the Single Carrier-frequency division multiple access (SC-FDMA) scheme [15]. Moreover, the signals transmitted from the MUE are detected by digital time delay estimation (TDE) receivers [16], utilizing a digital cross-correlation technique in the frequency domain. In the multi-FeNB TDOA positioning system proposed in this study, the received signal of each FeNB is sent through the Internet to a reference FeNB, which calculates the TDOA between itself and each FeNB such that the position of the MUE can be further derived.

Assume that the moving MUE emits an SC-FDMA signal s[*n*] in 3D space. The received signal at the reference FeNB (FeNB_0_) thus has the form:

(1) $$x_{0}[n]=s_{0}[n]+w_{0}[n],n\in [0,M-1]$$

where $w_{0}[n]$ is the discrete white Gaussian noise at FeNB_0_; and the variable *n* is defined as the sampling instant. The received signals at three other arbitrarily-chosen FeNBs are given by:

(2) $$\begin{array}{cc} x_{i}[n] & =s_{i}[n-D_{i0}]+w_{i}[n]\\ & \;n\in [0,M-1],\;i=1,2,3 \end{array}$$

where $w_{i}[n]$ is the discrete white Gaussian noise at FeNB*_i_*. The discrete time delay between FeNB*_i_* and FeNB_0_ is estimated as:

(3) $${\hat{l}}_{i}=D_{i0},\;i=1,2,3$$

Four-FeNB TDOA localization schemes calculate the possible MUE location based on the TDOA measurements of three FeNB receiver pairs lying on separate hyperbola [10]. In other words, the MUE location is obtained by solving three hyperbolic equations, i.e.:

(4) $$\begin{array}{cc} r_{i,0} & =\sqrt{{\left(\hat{x}-x_{0}\right)}^{2}+{\left(\hat{y}-y_{0}\right)}^{2}+{\left(\hat{z}-z_{0}\right)}^{2}}\\ & -\sqrt{{\left(\hat{x}-x_{i}\right)}^{2}+{\left(\hat{y}-y_{i}\right)}^{2}+{\left(\hat{z}-z_{i}\right)}^{2}};\;i=1,2,3\; \end{array}$$

where $\left(x_{i},y_{i},z_{i}\right)$ and $\left(x_{0},y_{0},z_{0}\right)$ are the coordinates of the ith and reference FeNBs, respectively; and $\left(\hat{x},\hat{y},\hat{z}\right)$ is the estimated MUE location. In addition, $r_{i,0}$ is the differential distance between the MUE and the master FeNB, and the MUE and FeNB*_i_*, respectively, and is obtained from the measured time delay between FeNB*_i_* and FeNB_0_ as:

(5) $$\begin{array}{l} r_{i,0}=\frac{cD_{i0}}{Mf_{s}}+e_{r_{i,0}}=c{\hat{\tau }}_{i,0}+e_{r_{i,0}}=c({\hat{\tau }}_{i}-{\hat{\tau }}_{0})+e_{r_{i,0}}\\ \;i=1,2,3 \end{array}$$

where *M* is the size of the FFT, ${\hat{\tau }}_{i,0}$ is the analog time delay between FeNB*_i_* and FeNB_0_, and $f_{s}$ is the sampling frequency of the analog-to-digital converter. Finally, $e_{r_{i,0}}$ is the measurement error with standard deviation $\sigma_{e_{i}}$.

The accuracy of range-based positioning location (PL) systems depends on the geometric relationship between the locations of the FeNBs and the location of the MUE, since errors arising from channel or hardware perturbations in the TDOA estimates are transformed into geographic position errors in the hyperbolic solution [10]. The positioning accuracy of different geometric FeNB configurations can be evaluated using the GDOP parameter, which is defined as the ratio of the root mean square (RMS) position error to the RMS ranging error. The GDOP for an unbiased estimator and a 3D hyperbolic system are given by [10]:

(6) $$\mathrm{GDOP}=\frac{1}{\sqrt{N}}\overset{N}{\underset{n=1}{\sum }}\frac{\sqrt{{\left(x-{\hat{x}}_{n}\right)}^{2}+{\left(y-{\hat{y}}_{n}\right)}^{2}+{\left(z-{\hat{z}}_{n}\right)}^{2}}}{\sigma_{e_{i}}}$$

where *N* is the number of measurements; $\sigma_{e_{i}}$ is the standard deviation of the measurement error $e_{r_{i,0}}$; and (x,y,z) and $\left({\hat{x}}_{n},{\hat{y}}_{n},{\hat{z}}_{n}\right)$ are the true location and the *n*th estimated location of the MUE, respectively. To calculate the GDOP, it is necessary to know the positions of the FeNBs around the moving MUE. A symbol table is shown in Table 1, which is used to keep track of the symbols and notations used in the paper.

## 3. Principle of NMLP Geometry Classifier

**Figure 2:**
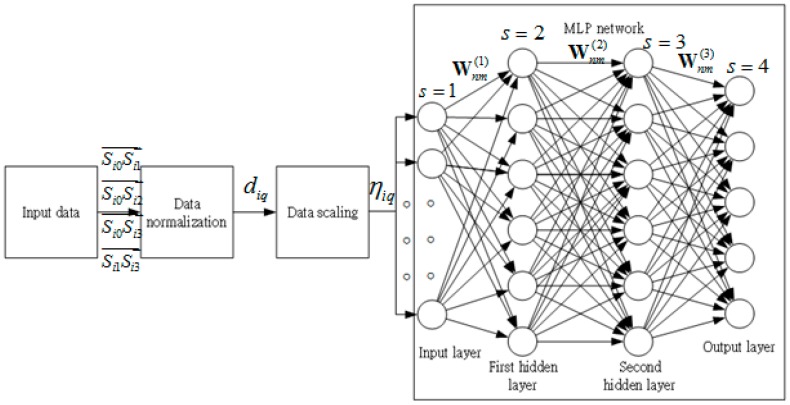
Architecture of the NMLP geometry classifier.

The architecture of the NMLP geometry classifier is shown in Figure 2. It consists of input data normalization, data scaling, an input layer, two hidden layers, and an output layer. The input vectors include:

(7) $$\begin{array}{l} \vec{s_{i0}s_{i1}}=(x_{i1}-x_{i0},y_{i1}-y_{i0},z_{i1}-z_{i0})\\ \vec{s_{i0}s_{i2}}=(x_{i2}-x_{i0},y_{i2}-y_{i0},z_{i2}-z_{i0})\\ \vec{s_{i0}s_{i3}}=(x_{i3}-x_{i0},y_{i3}-y_{i0},z_{i3}-z_{i0})\\ \vec{s_{i1}s_{i3}}=(x_{i3}-x_{i1},y_{i3}-y_{i1},z_{i3}-z_{i1}) \end{array}$$

where the coordinates of *P* set of the four FeNBs are $S_{i}=s_{i0}(x_{0},y_{0},z_{0})s_{i1}(x_{1},y_{1},z_{1})s_{i2}(x_{2},y_{2},z_{2})s_{i3}(x_{3},y_{3},z_{3});\;i\;=\;1,2,...,P.$, where $s_{ij}$ is the *j*th component of the *i*th input vector and *P* is the total number of four FeNBs in the coverage range of the target MUE. Note that each component in a vector of a quadrilateral is constituted by four FeNBs. The data normalization is obtained by:

(8) $$\begin{array}{l} {\vec{S_{i0}S_{i1}}}^{\prime }=\frac{\vec{S_{i0}S_{i1}}}{L_{i,\max}}=({\left(x_{i1}-x_{i0}\right)}^{\prime },{\left(y_{i1}-y_{i0}\right)}^{\prime },{\left(z_{i1}-z_{i0}\right)}^{\prime })=(d_{i1},d_{i2},d_{i3})\\ {\vec{S_{i0}S_{i2}}}^{\prime }=\frac{\vec{S_{i0}S_{i2}}}{L_{i,\max}}=({\left(x_{i2}-x_{i0}\right)}^{\prime },{\left(y_{i2}-y_{i0}\right)}^{\prime },{\left(z_{i2}-z_{i0}\right)}^{\prime })=(d_{i4},d_{i5},d_{i6})\\ {\vec{S_{i0}S_{i3}}}^{\prime }=\frac{\vec{S_{i0}S_{i3}}}{L_{i,\max}}=({\left(x_{i3}-x_{i0}\right)}^{\prime },{\left(y_{i3}-y_{i0}\right)}^{\prime },{\left(z_{i3}-z_{i0}\right)}^{\prime })=(d_{i7},d_{i8},d_{i9})\\ {\vec{S_{i1}S_{i3}}}^{\prime }=\frac{\vec{S_{i1}S_{i3}}}{L_{i,\max}}=({\left(x_{i3}-x_{i1}\right)}^{\prime },{\left(y_{i3}-y_{i1}\right)}^{\prime },{\left(z_{i3}-z_{i1}\right)}^{\prime })=(d_{i10},d_{i11},d_{i12}) \end{array}$$

where $L_{\max}=\mathrm{maximum}\;\mathrm{of}\{\left\|\vec{s_{i0}s_{i1}}\right\|,\left\|\vec{s_{i1}s_{i2}}\right\|,\left\|\vec{s_{i2}s_{i3}}\right\|,\left\|\vec{s_{i3}s_{i0}}\right\|\}.$

The scaled input and output data are required for the MLP, in order to provide the correct value for an activation function. The linear scaling of data is the transformation from the original data variability within the interval $\left[d_{q,\min},d_{q,\max}\right]$ to the interval [0.1, 0.99], and can be performed using the Formula [10]:

(9) $$\eta_{iq}=\frac{\left(d_{iq}-d_{q,\min})(b^{\prime }-a^{\prime }\right)}{d_{q,\max}-d_{q,\min}},\;q=1,...,Q;i=1,...,P$$

where $\left[a^{\prime },b^{\prime }\right]=\left[0.1,0.99\right].$ $d_{iq}$ is the original length of the *i*th input component at the *q*th neuron of the input layer. The MLP network of the geometry classifier is composed of 12 (Q) inputs, which represent 12 features of the NMLP geometry classifier.

The sigmoid activation function [12] used is non-linear, differentiable, and is defined by:

(10) $$y(a_{n}(k))=1/(1+e^{-\alpha a_{n}(k)})$$

the neuron outputs of last layer where $m_{l}=5$ is the total number of neurons in the last layer.

Given the scaled training data: ***D*** = {($S_{i1}(k),D_{i1}(k))$, ..., $\left(S_{im_{l}}(k),D_{im_{l}}(k)\right)$} where $S_{iq}(k),D_{iq}(k)$ are the given input and the desired output at the *k*th iteration.

The weight matrices are updated by:

(11) $$W_{nm}^{\left(s\right)}(q+1)=W_{nm}^{\left(s\right)}(q)+\mu^{\left(s\right)}(q)\vec{\delta_{n}^{\left(s\right)}(q)}\cdot \vec{x_{out,n}^{\left(s-1\right)}(q)}$$

where the learning rate is given by:

(12) $$\mu^{\left(v\right)}=\mu_{0}/(1+(k/t))\;(100<t<500)$$

For the hidden layer:

(13) $$\vec{\delta_{j}^{\left(s\right)}(q)}=(\overset{n_{s+1}}{\underset{h=1}{\Sigma }}\vec{\delta_{h}^{\left(s+1\right)}(q)}\cdot \vec{w_{hj}^{\left(s+1\right)}(q)})g(\vec{v_{j}^{\left(s\right)}(q)})$$

For the output layer:

(14) $$\vec{\delta_{j}^{\left(s\right)}(q)}=(\vec{d_{output}(q)}-\vec{x_{out,j}^{\left(s\right)}(q)})g(\vec{v_{j}^{\left(s\right)}(q)})$$

During the training, the network weights are adjusted in order to reduce the mean square error (MSE) obtained by [12]

(15) $$\mathrm{MSE}=\frac{\overset{m_{l}}{\underset{q=1}{\Sigma }}{\left(s_{iq}-d_{iq}\right)}^{2}}{m_{l}}$$

The performance of the NMLP geometry classifier will be evaluated in terms of the percentages for correct classification. It is defined on the difference between the desired output and the estimated output of the NMLP geometry classifier. The classification accuracy is defined as [12]:

(16) $$\mathrm{classification}\;\mathrm{accuracy}=\frac{N_{c}}{P}\times 100\%$$

where $N_{c}$ is the total number of correct classifications and *P* is the total number of four FeNBs within the sphere.

**Figure 3:**
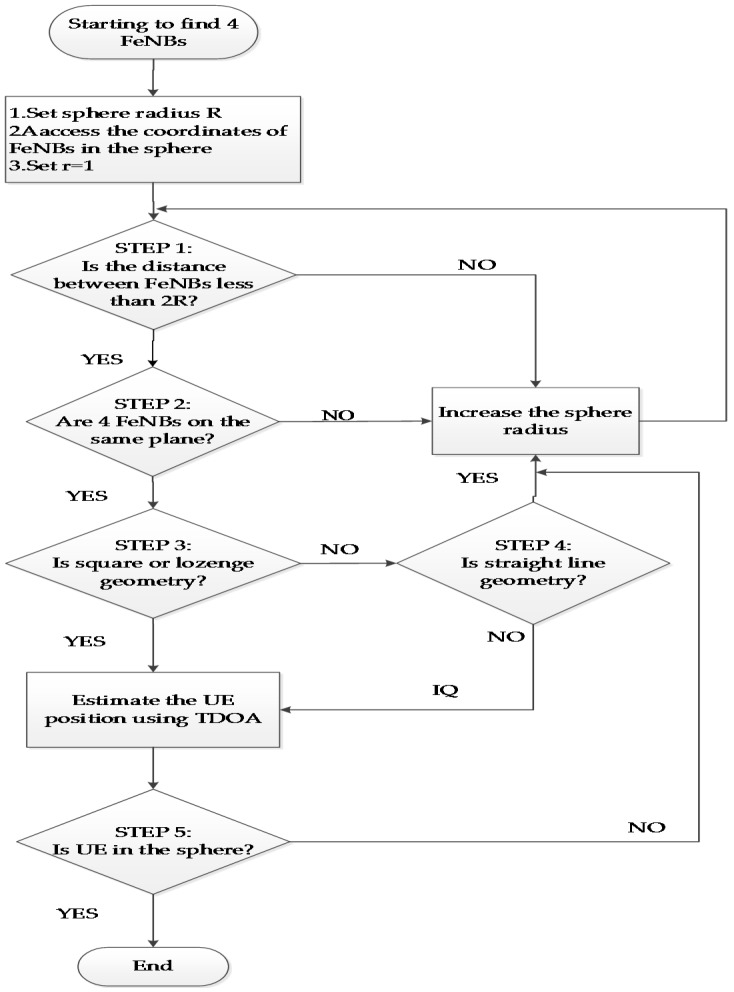
IGT procedure for generating the training data.

The flowchart of the IGT algorithm is shown in Figure 3, which is used to generate the training data for the NMLP geometry classifier. As shown in Figure 3, the IGT algorithm comprises five steps. In the first step, four FeNBs are chosen such that the distance between each pair of FeNBs is less than the pre-specified diameter of a sphere (expressed in meters). In other words, four FeNBs are selected such that the following equations are satisfied:

(17) $$\begin{array}{l} \sqrt{{\left(x_{i}-x_{0}\right)}^{2}+{\left(y_{i}-y_{0}\right)}^{2}+{\left(z_{i}-z_{0}\right)}^{2}}\leq 2R\;,\;i=1,2,3\\ \sqrt{{\left(x_{i}-x_{i+1}\right)}^{2}+{\left(y_{i}-y_{i+1}\right)}^{2}+{\left(z_{i}-z_{i+1}\right)}^{2}}\leq 2R\;,\;i=1,2\\ \sqrt{{\left(x_{i}-x_{i+2}\right)}^{2}+{\left(y_{i}-y_{i+2}\right)}^{2}+{\left(z_{i}-z_{i+2}\right)}^{2}}\leq 2R\;,\;i=1 \end{array}$$

where ${\left(x_{i},y_{i},z_{i}\right)}^{T}\;i=0,1,2,3$ are the coordinates of the four FeNBs; *R* is the sphere radius; and *T* denotes the transpose of the matrix. In the second step, the selected set of FeNBs is updated as required, to ensure that all four FeNBs are located on the same plane. Note that the sufficient condition for such a case occurs when the parallelepiped volume constructed by the three vectors $\vec{s_{0}s_{1}},\vec{s_{0}s_{2}},\vec{s_{0}s_{3}}$ is equal to zero. In other words:

(18) $$\left\|\begin{array}{ccc} x_{1}-x_{0} & y_{1}-y_{0} & z_{1}-z_{0}\\ x_{2}-x_{0} & y_{2}-y_{0} & z_{2}-z_{0}\\ x_{3}-x_{0} & y_{3}-y_{0} & z_{3}-z_{0} \end{array}\right\|=0$$

where the FeNB located at coordinates ${\left(x_{0},y_{0},z_{0}\right)}^{T}$ is chosen as the reference station. In the third step, the geometry disposition of the four FeNBs is evaluated to ensure that the FeNBs form either a square or a lozenge shape. In this case, the sufficient condition is taken as two of the vectors having a perpendicular orientation to one another. In other words, the inner product of the two vectors is equal to zero, i.e.:

(19) $$\vec{s_{0}s_{2}}\cdot \vec{s_{1}s_{3}}=0$$

In the fourth step, a check is made to confirm that the four FeNBs are not located on a straight-line.

If four FeNBs are located on a straight-line, this increases the radius, and if not, then the shape is an IQ. In general, two vectors are parallel if their outer product is equal to zero. Thus, the four FeNBs are not located on a straight-line if A∪B∪C is valid, where A, B, and C are defined, respectively, as follows:

(20) $$\begin{array}{l} A:\;\vec{s_{0}s_{1}}\times \vec{s_{0}s_{2}}\neq 0\\ B:\;\vec{s_{0}s_{1}}\times \vec{s_{0}s_{3}}\neq 0\\ C:\;\vec{s_{0}s_{2}}\times \vec{s_{0}s_{3}}\neq 0 \end{array}$$

If the conditions associated with Steps 1, 2, and 3 are satisfied, the optimal four-FeNB disposition has a low GDOP. Otherwise, if the conditions associated with Steps 1, 2, and 4 are satisfied, the acceptable four-FeNB configuration is determined with a higher GDOP. A lower GDOP represents a better localization result. As soon as either case is satisfied, the coordinates of the moving MUE are estimated using the TDOA method. Finally, in the fifth step, a check is made to confirm that the four FeNBs and MUE are located within the designated sphere, i.e.:

(21) $$\begin{array}{cc} {\left(x_{i}-\hat{x}\right)}^{2}+ & {\left(y_{i}-\hat{y}\right)}^{2}+{\left(z_{i}-\hat{z}\right)}^{2}\\ & \leq {\left[200+\left(r-1\right)\times 20\right]}^{2}=R^{2}\\ & \;i=0,1,2,3\;,\;r=1,2,3,\cdot \cdot \cdot \end{array}$$

where the coverage radius *R* of the MUE is initiated with 200 m and increased with the increment of 20 m.

## 4. Optimum Geometry Disposition Decision Criteria

It was noted that the optimum quadrilateral shapes, consisting of four FeNBs, can be selected to measure the location of the moving MUE. The square is the best, the lozenge is next, and the IQ is the worst. When two or more of the same quadrilateral shapes are selected, the following three optimum geometry disposition decision criteria are considered, to determine the best accuracy of the TDOA positioning measures. The GDOP formulas of three optimum geometry disposition decision criteria are derived. Since the location of four FeNBs is fixed, the quadrilateral shape of the FeNBs does not change due to the movement of the MUE. Therefore, in order to simplify the proof of selecting the optimum shapes of four FeNBs, three different criteria are proved in the two dimensional space, and the results are still valid in the three dimensional space.

Criterion 1: Square optimum geometry disposition decision criterion depends on the distance between the MUE and the square center. When the distance is shorter, the positioning accuracy is better.

Let the distance from the MUE to the *i*th FeNB be given by [11]:

(22) $$r_{i,TDOA}=\sqrt{{\left(x-x_{i}\right)}^{2}+{\left(y-y_{i}\right)}^{2}},\;i=0,1,2,3$$

The differential distance is obtained from subtracting two TDOA measurements between FeNB*_i_* and FeNB_0_.

(23) $$\Delta r_{i,TDOA}=r_{i,TDOA}-r_{0,TDOA},\;i=1,2,3$$

The TDOA measurements for three pairs of FeNBs are given as:

(24) $$t_{i}-t_{0}=\frac{\Delta r_{i,TDOA}}{c}+\frac{\left(\varepsilon_{i}-\varepsilon_{0}\right)}{c}i=1,2,3$$

where *c* is the light velocity and the measurement error is expressed as:

(25) $$\varepsilon =\left[\begin{array}{c} \varepsilon_{0}\\ \varepsilon_{1}\\ \varepsilon_{2}\\ \varepsilon_{3} \end{array}\right]$$

It is assumed that the errors in all actual measurements are random, independent, and have an identical root mean square (RMS) value $\sigma_{r}^{2}$.

(26) $$\begin{array}{l} E\left[\varepsilon_{i}\right]=\bar{\varepsilon }\\ E\left[\varepsilon_{i}\varepsilon_{j}\right]=0\\ E\left[\varepsilon_{i}^{2}\right]=\sigma_{r}^{2}+{\bar{\varepsilon }}^{2},i=0,1,2,3\\ \sigma_{r}^{2}=E\left[{\left(\varepsilon_{i}-E\left[\varepsilon_{i}\right]\right)}^{2}\right]=E\left[\varepsilon_{i}^{2}\right]-{\bar{\varepsilon }}^{2} \end{array}$$

The TDOA measurement errors for three pairs of FeNBs can be expressed as the matrix form.

(27) $$\left[\begin{array}{c} \left(\varepsilon_{1}-\varepsilon_{0}\right)\\ \left(\varepsilon_{2}-\varepsilon_{0}\right)\\ \left(\varepsilon_{3}-\varepsilon_{0}\right) \end{array}\right]=\left[\begin{array}{l} \begin{array}{cccc} -1 & 1 & 0 & 0 \end{array}\\ \begin{array}{cccc} -1 & 0 & 1 & 0 \end{array}\\ \begin{array}{cccc} -1 & 0 & 0 & 1 \end{array} \end{array}\right]\left[\begin{array}{c} \varepsilon_{0}\\ \varepsilon_{1}\\ \varepsilon_{2}\\ \varepsilon_{3} \end{array}\right]=A\varepsilon$$

The error covariance matrix is:

(28) $$\begin{array}{cc} Q & =E\left[(A\varepsilon ){\left(A\varepsilon \right)}^{T}\right]=AE\left[\varepsilon \varepsilon^{T}\right]A^{T}=({\sigma_{r}}^{2}+{\bar{\varepsilon }}^{2})AA^{T}\\ & =(\sigma_{r}^{2}+{\bar{\varepsilon }}^{2})\left[\begin{array}{ccc} 2 & 1 & 1\\ 1 & 2 & 1\\ 1 & 1 & 2 \end{array}\right] \end{array}$$

The partial derivatives of the noise free measurement Equations (23) with respect to the unknown MUE coordinates (*x*, *y*) are given as:

(29) $$d\Delta r_{i}=(\frac{x-x_{i}}{r_{i,TDOA}}-\frac{x-x_{0}}{r_{0,TDOA}})dx+(\frac{y-y_{i}}{r_{i,TDOA}}-\frac{y-y_{0}}{r_{0,TDOA}})dy;\;i=1,2,3$$

The matrix is expressed as:

(30) $$\left[\begin{array}{l} d\Delta r_{1,TDOA}\\ d\Delta r_{2,TDOA}\\ d\Delta r_{3,TDOA} \end{array}\right]=\left[\begin{array}{l} \frac{x-x_{1}}{r_{1,TDOA}}-\frac{x-x_{0}}{r_{o,TDOA}}+\frac{y-y_{1}}{r_{1,TDOA}}-\frac{y-y_{0}}{r_{o,TDOA}}\\ \frac{x-x_{2}}{r_{2,TDOA}}-\frac{x-x_{0}}{r_{o,TDOA}}+\frac{y-y_{2}}{r_{2,TDOA}}-\frac{y-y_{0}}{r_{o,TDOA}}\\ \frac{x-x_{3}}{r_{3,TDOA}}-\frac{x-x_{0}}{r_{o,TDOA}}+\frac{y-y_{3}}{r_{3,TDOA}}-\frac{y-y_{0}}{r_{o,TDOA}} \end{array}\right]\left[\begin{array}{l} dx\\ dy \end{array}\right]$$

where:

(31) $$H=\left[\begin{array}{c} \frac{x-x_{1}}{r_{1,TDOA}}-\frac{x-x_{0}}{r_{o,TDOA}}\\ \frac{x-x_{2}}{r_{2,TDOA}}-\frac{x-x_{0}}{r_{o,TDOA}}\\ \frac{x-x_{3}}{r_{3,TDOA}}-\frac{x-x_{0}}{r_{o,TDOA}} \end{array}\begin{array}{c} \frac{y-y_{1}}{r_{1,TDOA}}-\frac{y-y_{0}}{r_{o,TDOA}}\\ \frac{y-y_{2}}{r_{2,TDOA}}-\frac{y-y_{0}}{r_{o,TDOA}}\\ \frac{y-y_{3}}{r_{3,TDOA}}-\frac{y-y_{0}}{r_{o,TDOA}} \end{array}\right]$$

When the error covariance matrix is not diagonal, as in Equation (28), the GDOP will be given by:

(32) $$\mathrm{GDOP}=\sqrt{trace(G)}$$

where the matrix ***G*** is the variance of the linear optimum unbiased estimator divided by the variance of measurement noise [17].

(33) $$G=\frac{1}{{\sigma_{r}}^{2}}{\left(H^{T}Q^{-1}H\right)}^{-1}$$

For the simplification of proving the square optimum geometry disposition decision criterion, the coordinates of FeNB_0_ (1, 0), FeNB_1_ (0, 1), FeNB_2_ (−1, 0), FeNB_3_ (0, −1), and MUE (*x*, *y*) are substituted into Equation (29), to yield:

(34) $$H=\left[\begin{array}{c} \frac{x}{\sqrt{x^{2}+{\left(y-1\right)}^{2}}}-\frac{x-1}{\sqrt{{\left(x-1\right)}^{2}+y^{2}}}\\ \frac{x+1}{\sqrt{{\left(x+1\right)}^{2}+y^{2}}}-\frac{x-1}{\sqrt{{\left(x-1\right)}^{2}+y^{2}}}\\ \frac{x}{\sqrt{x^{2}+{\left(y+1\right)}^{2}}}-\frac{x-1}{\sqrt{{\left(x-1\right)}^{2}+y^{2}}} \end{array}\begin{array}{c} \frac{y-1}{\sqrt{x^{2}+{\left(y-1\right)}^{2}}}-\frac{y}{\sqrt{{\left(x-1\right)}^{2}+y^{2}}}\\ \frac{y}{\sqrt{{\left(x+1\right)}^{2}+y^{2}}}-\frac{y}{\sqrt{{\left(x-1\right)}^{2}+y^{2}}}\\ \frac{y+1}{\sqrt{x^{2}+{\left(y+1\right)}^{2}}}-\frac{y}{\sqrt{{\left(x-1\right)}^{2}+y^{2}}} \end{array}\right]$$

**Figure 4:**
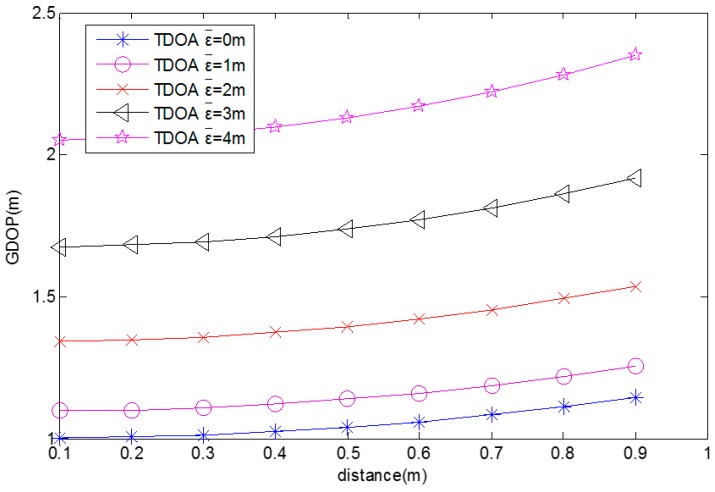
GDOP for different distances.

When the MUE moves from (1, 0) to (0.1, 0), the GDOPs for different average TDOA position measurement errors (0, 1, 2, 3, 4 m) are computed and these are shown in Figure 4. It shows that the GDOP decreases with the distance between the MUE and the square center. The GDOP also decreases with the average TDOA position measurement error.

Criterion 2: Lozenge optimum geometry disposition decision criterion depends on the complementary angle difference (CAD), which is the difference between two complementary angles of a lozenge shape. When the CAD of the lozenge optimum geometry disposition is smaller, the positioning accuracy is better.

For simplification, the GDOP formula is derived by substituting the coordinates of FeNB_0_ (1, 0), FeNB_2_ (−1, 0), FeNB_1_ (0, *N*), FeNB_3_(0, −*N*), and MUE (0.1, 0) into Equation (31), to yield:

(35) $$H=\left[\begin{array}{cc} \begin{array}{c} \frac{1}{10\sqrt{N^{2}+0.01}}+1\\ 2\\ \frac{1}{10\sqrt{N^{2}+0.01}}+1 \end{array} & \begin{array}{c} \frac{-N}{\sqrt{N^{2}+0.01}}\\ 0\\ \frac{N}{\sqrt{N^{2}+0.01}} \end{array} \end{array}\right]$$

Substituting Equations (28) and (35) into Equation (33) yields:

(36) $$G=\left[\begin{array}{cc} \frac{\left(5+{\bar{\varepsilon }}^{2})(N^{2}+0.01\right)}{200N^{2}+3} & 0\\ 0 & \frac{\left(5+{\bar{\varepsilon }}^{2})(N^{2}+0.01\right)}{10N^{2}} \end{array}\right]$$

Then the GDOP of lozenge shape is expressed as:

(37) $$\begin{array}{cc} GDOP & =G_{1,1}+G_{2,2}\\ & =\frac{\left({\bar{\varepsilon }}^{2}+5)(N^{2}+0.01\right)}{200N^{2}+3}+\frac{\left({\bar{\varepsilon }}^{2}+5)(N^{2}+0.01\right)}{10N^{2}} \end{array}$$

The CAD formula is derived as:

(38) $$\mathrm{CAD}=2(\arccos(\frac{1}{\sqrt{1+N^{2}}})-\arccos(\frac{N}{\sqrt{1+N^{2}}}))$$

**Figure 5:**
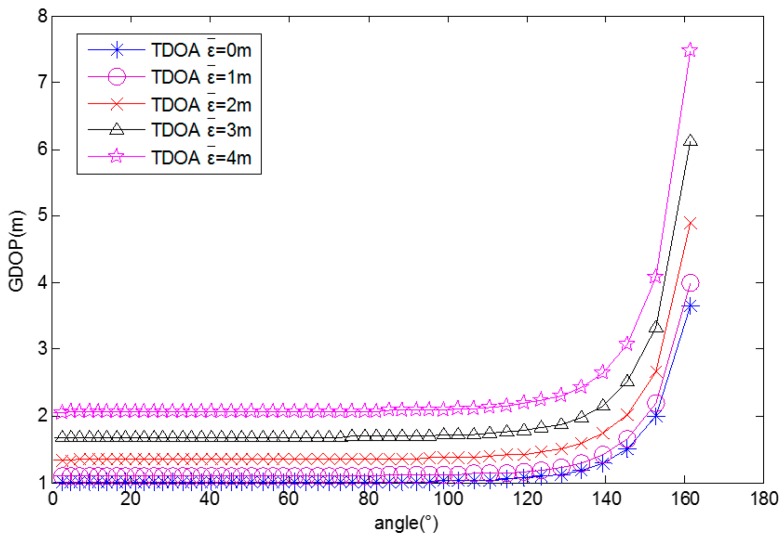
GDOP for different CADs.

Figure 5 is generated from Equations (37) and (38), where *N* varies from 0 to 1 and $\sigma_{r}^{2}$ = 5. It shows that the GDOP decreases with the CAD. The GDOP also decreases with the average TDOA position measurement error. When *N* = 1, CAD = 0°. This is the square shape, which has a smaller GDOP value than the lozenge shape. When N = 0, CAD = 180°. This is the straight line, which has the largest GDOP value.

Criterion 3: Irregular-quadrilateral optimum geometry disposition decision criterion depends on the distance between the diagonal intersection and the center of the IQ. When the distance is shorter, the positioning accuracy of the IQ optimum geometry disposition is better. 

For simplification, the GDOP formula is derived by substituting the coordinates of FeNB_0_ (0.5, 0), FeNB_1_ (0, $y_{1}$), FeNB_2_ (−0.5, 0), FeNB_3_ (0, −1), and MUE (0.1, 0) into Equation (31), to yield:

(39) $$H=\left[\begin{array}{cc} \begin{array}{c} \frac{1}{10\sqrt{{y_{1}}^{2}+0.01}}+\frac{9}{4}\\ 4.083\\ 2.3495 \end{array} & \begin{array}{c} \frac{-y_{1}}{\sqrt{{y_{1}}^{2}+0.01}}\\ 0\\ 0.995 \end{array} \end{array}\right],$$

**Figure 6:**
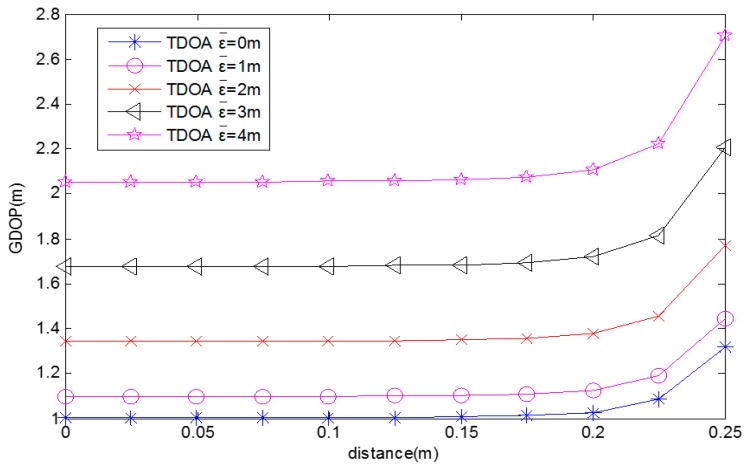
GDOP for different distances.

Figure 6 is generated from Equations (28), (32), (33), and (39), where $y_{1}$ varies from 0 to 1. It shows that the GDOP decreases with the distance between the diagonal intersection and the center of the quadrilateral and the average TDOA position measurement error.

## 5. Simulations 

In performing the simulations, it was assumed that the time delay between FeNB*_i_* and FeNB_0_ was successfully measured with a detection probability of P_d_ = 0.9, while the false alarm probability was equal to P_FA_ = 10^−4^. Moreover, the signal-to-noise ratio (SNR) of the TDE receivers with a linear optimum filter was assumed to be −13 dB over the Rayleigh channels, with P_d_ = 0.9 and P_FA_ = 10^−4^. The simulations considered the outdoor geo-location scenario shown in Figure 1, consisting of 16 FeNBs located in adjacent multi-floor buildings. The total number of four FeNBs in the coverage range of the target MUE is *P* = 1820. Moreover, the moving MUE was assumed to be located between two buildings and positioned at the boundary of a macrocell network. The coordinates of the MUE trajectory are (60, 0, 0), (60, 10, 0), (60, 20, 0), (60, 30, 0), (60, 40, 0), (60, 50, 0), (60, 60, 0), (60, 70, 0), (60, 80, 0), and (60, 90, 0). In simulations, the SC-FDMA signal was considered as it is used by LTE MUE as an uplink access scheme [7,15]. The SC-FDMA waveforms are specified with bandwidth 10 MHz, subcarrier spacing 15 KHz, FFT/IFFT size 1024, and preamble length 320. The sixteen FeNBs in accordance with their coordinate information are shown in Table 2. (Note that the coordinates are expressed in units of meters.) Any four FeNBs constituted a quadrilateral group. Moreover, the MUE is assumed to be moving between two adjacent buildings. The simulation tool is Matlab. After the simulations, the performance of the proposed NMLP geometry classifier using the multi-station TDOA MUE localization scheme was numerically evaluated.

The simulations are performed as follows. First, the IGT algorithm is used to calculate the training data of all quadrilateral FeNBs. Then, the NMLP geometry classifier is trained to determine the optimal shape by searching all of the quadrilateral sets of FeNBs within the detection range of the MUE signal. The weight matrices of MLP networks for the first, second, and third layers are $W_{nm}^{\left(2\right)}$, $W_{nm}^{\left(3\right)}$, and $W_{nm}^{\left(4\right)}$, respectively. The matrix dimensions are 12 × 6, 6 × 6 and 6 × 5, respectively. The desired outputs obtained from the training results are classified as five different shapes, which include [0.99, 0.01, 0.01, 0.01, 0.01], [0.01, 0.99, 0.01, 0.01, 0.01], [0.01, 0.01, 0.99, 0.01, 0.01], [0.01, 0.01, 0.01, 0.99, 0.01], and [0.01, 0.01, 0.01, 0.01, 0. 99] for the square, lozenge, IQ, straight-line, and non-planar shapes, respectively. Finally, the optimum geometry disposition decision criteria are used to determine the best accuracy of the TDOA positioning measures.

**Figure 7:**
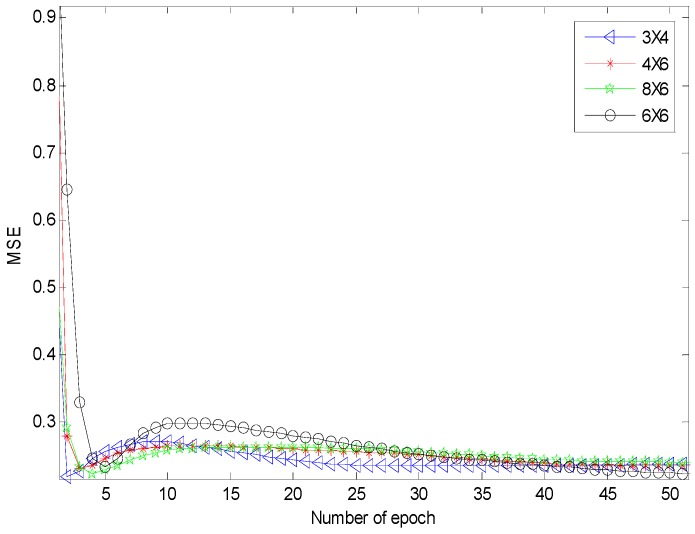
MSE of the MLP network using a different number of neurons for the first and second layers.

**Figure 8:**
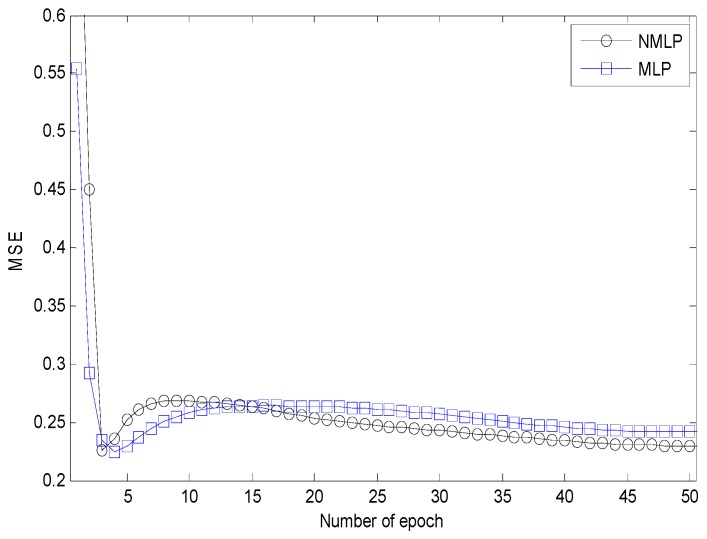
MSE comparison between NMLP and MLP networks for six by six neurons in the first and second layers.

Figure 7 shows that the MLP network using six by six neurons for the hidden layers has the shortest convergence time. It achieved a classification accuracy of 98.0659%. Figure 8 shows that the NMLP geometry classifier can improve the convergent time, and the classification accuracy achieved was 98.0330%. Table 3 shows the estimated NMLP network outputs of five neurons for five different shapes. The estimated NMLP geometry classifier outputs are [0.9418, 0.0186, 0.0587, 0.0145, 0.0173], [0.0504, 0.8951, 0.0751, 0.0291, 0.0912], [0.0124, 0.0186, 0.9427, 0.0624, 0.0174], [0.0107, 0.0181, 0.0114, 0.9755, 0.0130], and [0.0178, 0.0136, 0.0584, 0.0175, 0.9581] which are classified as square, lozenge, IQ, straight-line, and non-planar shapes, respectively.

The classification results after training are shown in Table 4, which shows the number of geometry classifications for three cases. For all FeNBs, a unique square geometry is selected by the proposed NMLP geometry classifier to estimate the current MUE position obtained using the TDOA scheme. Here, the TDOA measurement error of each FeNB is assumed to be Gaussian distributed with a mean of zero and a standard deviation of $\sigma_{e_{i}}=5\;m$. Moreover, the true coordinates of the MUE are assumed to be (60, 50, 0). The GDOP value is calculated as 1.533 m. For the case not including FeNB_4_ in the adjacent multi-floor buildings, *P* reduces to 1465, and two lozenge geometry groups are selected by the proposed NMLP geometry classifier. The CAD of the first lozenge geometry group is 0.8° and the CAD of the second lozenge group is 3.1°. The GDOP value of the former group is 2.012 m and the GDOP value of the latter group is 2.432 m. Then, four FeNBs of a lozenge geometry with a GDOP value of 2.012 m are determined by the proposed NMLP geometry classifier. The decision criterion 2 is verified. The positioning accuracy is better when the CAD of the lozenge optimum geometry disposition is smaller. The TDOA measurement error of the lozenge geometry group is larger than the square geometry group if the FeNB_4_ is not deployed in the adjacent multi-floor buildings. For the case not including FeNB_4_ and FeNB_8_ in the adjacent multi-floor buildings, *P* reduces to 1001, and all of the square and lozenge geometry groups will be removed. In accordance with the IQ optimum geometry disposition decision criterion, the IQ group with the minimum GDOP value of 4.354 will be selected from 263 IQ groups.

When the MUE moves from the position (60, 0, 0) to (60, 90, 0), the trajectory of the moving path is shown in Figure 1. The proposed NMLP geometry classifier determines the first three best shapes for the three cases. The GDOP values of the first three best quadrilateral shapes for all FeNBs are listed in Table 5, which shows that the GDOP of the square geometry group is smaller when the distance between the MUE and the square center is shorter. The GDOP of the square geometry group is smaller than the lozenge geometry. When two lozenge geometry groups are selected by the proposed NMLP geometry classifier, the GDOP for the CAD of 0.8° is smaller than the CAD of 3.1°. In this case, the former is determined. Both decision criteria 1 and 2 are verified by the simulation results of Table 5. Table 6 shows the GDOP values of the first three best quadrilateral shapes for the case not including FeNB_4_ in the adjacent multi-floor buildings. It shows that the GDOP of the lozenge geometry group is smaller than the IQ geometry. The GDOP values of the first three best quadrilateral shapes for the case not including FeNB_4_ and FeNB_8_ in the adjacent multi-floor buildings are shown in Table 7, which demonstrates that the GDOP values are smaller when the distance between the diagonal intersection and the center of the IQ is shorter. The decision criterion 3 is verified. When four FeNBs with coordinates (0, 0, 50), (0, 180, 50), (180, 0, 50), and (180, 180, 50) are added into the buildings, the total number of four FenBs in the coverage range of the target MUE increases to *P* = 4845. The simulation results show that the GDOP value is calculated as 1.053 m and the classification accuracy achieved is 97.196%.

## 6. Conclusions

This paper proposes a novel NMLP geometry classifier and IGT algorithm for autonomously reducing the positioning error of four-FeNB TDOA measurements. Three optimum geometry disposition decision criteria are analyzed for the square, lozenge, and other IQ shape of the four FeNBs, for positioning the moving MUE. The derived analytical expressions are generally applicable to geometries where the MUE is surrounded by the selected FeNBs. The simulation results have confirmed that the proposed NMLP geometry classifier and optimum geometry disposition decision criteria can provide accurate outdoor geo-location information on the MUE for indoor femtocells to support its quality of services in HetNet. A comparison of the analytical results with simulations using the typical geometries of outdoor positioning systems shows good agreement.

The computational burden is mainly caused by the offline IGT algorithm simulations pertaining to the construction of the training data. Once the initial learning of the NMLP is finished using the collected training data to optimize network weights based on the NMLP network outputs of five neurons for five different shapes, the additional computation overhead in NMLP is light. Since the operations of the NMLP geometry classification are executed in the server, it is assumed that the computation ability of the server is powerful enough to determine the optimal geometry disposition for femtocell positioning networks. The information regarding the training data that governs the NMLP geometry classification operation of femtocell positioning networks is stored in a database. As soon as a new operation condition occurs, the new training data is generated from the simulation and included in the database to rerun the IGT training procedure of Figure 3. The simulation results show that the proposed method is particularly suitable for the application of MUE positioning with a huge number of FeNBs.

How to optimally determine four FeNBs to position the UE in indoor scenarios is another hot research topic in current telecommunication industries and academics. The proposed NMLP geometry classifier and IGT algorithm will be applied to the study of indoor femtocell positioning networks.

## Figures and Tables

**Table 1:** Symbol table.

$e_{r_{i,0}}$	Measurement error
$\sigma_{e_{i}}$	Standard deviation of the measurement error
$w_{i}[n]$	Discrete white Gaussian noise
$r_{i,0}$	The differential distance between the MUE and the master FeNB, and the MUE and FeNB*_i_*
${\hat{\tau }}_{i,0}$	Analog time delay between FeNB*_i_* and FeNB_0_
$f_{s}$	Sampling frequency of the analog-to-digital converter
$W_{nm}^{\left(s\right)}(q)$	Weight
$\mu_{0}$	Learning rate
c	Light velocity
*G*	The variance of the linear optimum unbiased estimator divided by the variance of measurement noise
ε	Measurement error
*R*	Sphere radius
*T*	Transpose of the matrix
$N_{c}$	Total number of correct classifications
*P*	Total number of 4 FeNBs within the sphere

**Table 2:** Coordinates of sixteen FeNBs.

FeNB_1_: (0, 0, 30)	FeNB_5_: (0, 0, 40)
FeNB_2_: (0, 120, 30)	FeNB_6_: (0, 120, 60)
FeNB_3_: (120, 0, 30)	FeNB_7_: (120, 0, 20)
FeNB_4_: (120, 120, 30)	FeNB_8_: (120, 120, 40)
FeNB_9_: (0, 60, 5)	FeNB_13_: (0, 0, 20)
FeNB_10_: (0, 60, 60)	FeNB_14_: (0, 15, 30)
FeNB_11_: (120, 0, 0)	FeNB_15_: (0, 30, 40)
FeNB_12_: (120, 100, 60)	FeNB_16_: (0, 45, 50)

**Table 3:** Estimated NMLP output for different geometries.

	Neuron 1 Output	Neuron 2 Output	Neuron 3 Output	Neuron 4 Output	Neuron 5 Output
square	0.9418	0.0186	0.0587	0.0145	0.0173
lozenge	0.0504	0.8951	0.0751	0.0291	0.0912
IQ	0.0124	0.0186	0.9427	0.0624	0.0174
straight line	0.0107	0.0181	0.0114	0.9755	0.0130
not co-plane	0.0178	0.0136	0.0584	0.0175	0.9581

**Table 4:** Number of geometry classifications.

	Square	Lozenge	IQ	Straight Line	Not Co-Plane
all FeNBs	1	6	217	4	1593
FeNB 4 not included	0	2	139	4	1220
FeNB4,8 not included	0	0	263	5	733

**Table 5:** GDOP of the first three best shapes for all FeNBs.

	CAD	Square (0° CAD) (Distance of MUE to Square Center)	Lozenge (0.8° CAD)	Lozenge (3.1° CAD)
	MUE Trajectory			
(60, 0, 0)		8.114 (67.082 m)	10.901	12.648
(60, 10, 0)		6.359 (58.31 m)	7.506	9.796
(60, 20, 0)		4.781 (50 m)	6.905	7.203
(60, 30, 0)		3.203 (42.426 m)	4.631	5.049
(60, 40, 0)		1.806 (36.056 m)	2.564	3.593
(60, 50, 0)		1.533 (31.623 m)	2.012	2.432
(60, 60, 0)		1.053 (30 m)	1.435	1.915
(60, 70, 0)		1.546 (31.623 m)	2.216	2.609
(60, 80, 0)		2.371 (36.056 m)	2.651	3.140
(60, 90, 0)		2.689 (42.426 m)	3.206	3.710

**Table 6:** GDOP of the first three best shapes not including FeNB_4_.

	CAD	Lozenge (0.8° CAD)	Lozenge (3.1° CAD)	IQ (0 m)
MUE Trajectory				
(60, 0, 0)		10.901	12.648	13.703
(60, 10, 0)		7.506	9.796	11.27
(60, 20, 0)		6.905	7.203	9.656
(60, 30, 0)		4.631	5.049	6.157
(60, 40, 0)		2.564	3.593	4.658
(60, 50, 0)		2.012	2.432	3.604
(60, 60, 0)		1.435	1.915	2.161
(60, 70, 0)		2.216	2.609	2.958
(60, 80, 0)		2.651	3.140	3.411
(60, 90, 0)		3.206	3.710	3.907

**Table 7:** GDOP of the first three best shapes not including FeNB_4_, FeNB_8_.

	Distance	IQ (0 m)	IQ (8.75 m)	IQ (15.052 m)
MUE Trajectory				
(60, 0, 0)		15.474	16.466	17.476
(60, 10, 0)		12.862	14.911	16.143
(60, 20, 0)		10.781	11.168	12.569
(60, 30, 0)		6.656	7.114	7.572
(60, 40, 0)		5.005	6.436	6.632
(60, 50, 0)		4.354	5.444	6.733
(60, 60, 0)		3.015	4.064	6.256
(60, 70, 0)		3.482	3.924	4.719
(60, 80, 0)		3.815	4.327	6.191
(60, 90, 0)		3.252	5.235	5.592

## References

[1] Cimmino A., Pecorella T., Fantacci R., Granelli F., Rahman T.F., Sacchi C., Carlini C., Harsh P. (2013). The Role of Small Cell Technology in Future Smart City Applications. Trans. Emerg. Telecommun. Technol..

[2] Boudreau G., Panicker J., Guo N., Wang N., Vrzic S. (2009). Interference Coordination and Cancellation for 4G Networks. IEEE Commun. Mag..

[3] Del Peral-Rosado J.A., Bavaro M., Lopez-Salcedo J.A., Seco-Granados G., Chawdhry P., Fortuny-Guasch J., Crosta P., Zanier F., Crisci M. Floor Detection with Indoor Vertical Positioning in LTE Femtocell Networks. Proceedings of the 2015 IEEE Globlecom Workshops.

[4] Wang C.-X., Haider F., Gao X., Yang Y., Yuan D., Aggoune H., Haas H., Fletcher S., Hepsaydir E. (2014). Cellular Architecture and Key Technologies for 5G Wireless Communication Networks. IEEE Commun. Mag..

[5] (2014). A 3GPP Description Document-Overview of 3GPP Release 9. www.3gpp.org.

[6] Zhang J., de la Roche G. (2010). Femtocells: Technologies and Deployment.

[7] Sesia S., Toufik I., Baker M. (2009). LTE: The UMTS Long Term Evolution: From Theory to Practice.

[8] 3GPP TS 36.305 (2010). Stage 2 Functional Specification of UE Positioning in E-UTRAN, Release 9.

[9] Johansson J., Hapsari W.A., Kelley S., Bodog G. (2012). Minimization of Drive Tests in 3GPP Release 11. IEEE Commun. Mag..

[10] Levanon N. (2000). Lowest GDOP in 2-D scenarios. IEE Proc. Radar.

[11] Isa I.S., Saad Z., Omar S., Osman M.K., Ahmad K.A., Mat Sakim H.A. Suitable MLP Network Activation Functions for Breast Cancer and Thyroid Disease Detection. Proceedings of the 2010 IEEE Second International Conference Computational Intelligence, Modeling and Simulation.

[12] Kasevski M., Tiwsin V., Pozdukhy A. (2009). Machine Learning for Spatial Environment Data, Theory, Application, and Sofware.

[13] Liberti J.C. Jr., Rappaport T.S. (1999). Smart Antennas for Wireless Communications: IS-95 and Third Generation CDMA Applications.

[14] Manolakis D.G., Ingle V.K., Kogon S.M. (2000). Statistical and Adaptive Signal Processing.

[15] Myung H.G., Goodman D.J. (2008). Single Carrier FDMA, a New Air Interface for Long Term Evolution.

[16] Chen J., Huang Y., Benesty J. (2004). Audio Signal Processing for Next-Generation Multimedia Communication System.

[17] Kay S.M. (1993). Fundamentals of Statistical Signal Processing: Estimation Theory.

